# Genetic Exploration of the Exit from Self-Renewal Using Haploid Embryonic Stem Cells

**DOI:** 10.1016/j.stem.2013.12.008

**Published:** 2014-03-06

**Authors:** Martin Leeb, Sabine Dietmann, Maike Paramor, Hitoshi Niwa, Austin Smith

**Affiliations:** 1 Wellcome Trust – Medical Research Council Stem Cell Institute, University of Cambridge, Tennis Court Road, CB2 1QR, Cambridge, UK; 2 Department of Biochemistry, University of Cambridge, Tennis Court Road, CB2 1GA, Cambridge, UK; 3 RIKEN Center for Developmental Biology, 650-0047 Kobe, Japan

## Abstract

Self-renewal circuitry in embryonic stem cells (ESCs) is increasingly defined. How the robust pluripotency program is dissolved to enable fate transition is less appreciated. Here we develop a forward genetic approach using haploid ESCs. We created libraries of transposon integrations and screened for persistent self-renewal in differentiation-permissive culture. This yielded multiple mutants in the Fgf/Erk and GSK3/Tcf3 modules known to drive differentiation and in epigenetic modifiers implicated in lineage commitment. We also identified and validated factors not previously considered. These include the conserved small zinc finger protein Zfp706 and the RNA binding protein Pum1. Pum1 targets several mRNAs for naive pluripotency transcription factors and accelerates their downregulation at the onset of differentiation. These findings indicate that the dismantling of pluripotent circuitry proceeds at multiple levels. More broadly they exemplify the power of haploid ESCs for genetic interrogation of developmental processes.

## Highlights

• We used haploid ESCs to develop a forward genetics screen for pluripotency regulators
• A cohort of candidate genes that promote collapse of self-renewal was validated
• The conserved zinc finger protein Zfp706 promotes differentiation
• Pum1 mediates posttranscriptional destabilization of the pluripotency network

## Graphical Abstract

**Figure:**
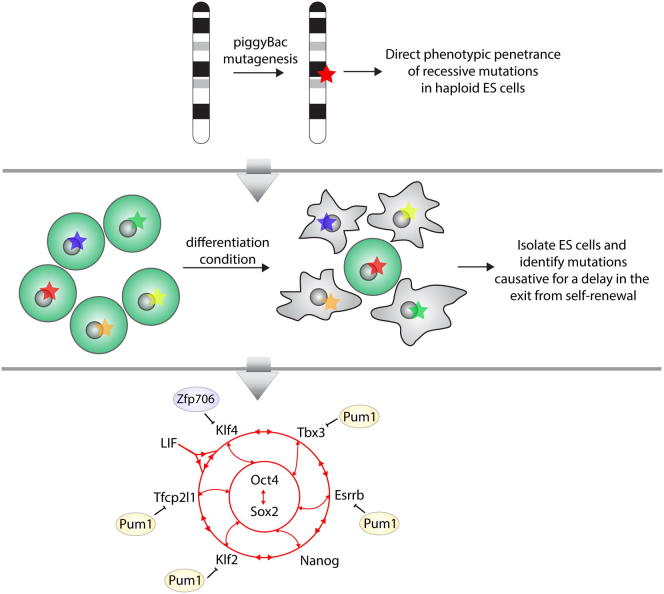

## Teaser

Leeb et al. identify key players regulating exit from self-renewal in ESCs using a forward genetic screen in haploid ESCs.

## Introduction

Rodent ESCs exhibit the identity and pluripotency of naive preimplantation epiblast cells with the additional attribute of extended self-renewal (Nichols and Smith, 2012). The molecular machinery and underlying genetic circuitry that sustain ESC character during self-renewal have been extensively characterized (Young, 2011). Less studied is the process by which ESCs exit the naive state to embark upon differentiation. In contrast to the ordered program of germ layer segregation that unfolds deterministically in the embryo and is obeyed by ESCs in chimeras, differentiation in vitro is asynchronous and disorganized (Lowell et al., 2006). Identifying factors and pathways that direct developmental progression from self-renewal to lineage commitment is a challenge. A timely opportunity for application of forward genetics to dissect this complex developmental transition arises from the recent derivation of haploid mouse ESCs (Elling et al., 2011; Leeb and Wutz, 2011).

Haploid ESCs can be derived from parthenogenetic embryos generated following chemical activation of unfertilized MII oocytes. Based on molecular marker analysis and gene expression profiles, haploid ESCs cannot be distinguished from their diploid counterparts. Notably, they retain full developmental potential and give rise to germline-competent chimeras (Leeb et al., 2012). Haploid ESCs are prone to diploidization in culture but can be maintained by periodic flow cytometric purification. Mutagenesis of the haploid genome allows recessive phenotypes to be directly unmasked. Proof of principle has been shown by screens to identify mutations that confer resistance to toxic compounds (Elling et al., 2011; Leeb and Wutz, 2011). Therefore haploid ESCs could provide a powerful system for elucidating the genetic circuitry of mammalian developmental processes.

Suppression of differentiation is sufficient to allow ESC self-renewal. This can be achieved by application of two small molecules (2i) that block the inductive stimulus of fibroblast growth factor 4 (Ffg4)/mitogen activated protein kinase (MAPK) signaling and partially inhibit glycogen synthase kinase-3 (GSK3) (Ying et al., 2008). 2i may capture ESCs in a “ground state” of self-renewal by insulating the core pluripotency transcription factor circuit (Nichols and Smith, 2012). Consistent with this idea, deficiency in components that promote collapse of the pluripotency network liberates self-renewal from a requirement for 2i (Betschinger et al., 2013; Wray et al., 2011).

Importantly, capacity for proliferation in 2i is rather specific for undifferentiated ESCs and is lost early in differentiation (Betschinger et al., 2013). Thus, the ability to self-renew in 2i after a period of permissive culture provides a powerful means to identify and quantify delayed exit from the ground state. Here we combine this functional assay together with haploid ESC mutagenesis in a genetic screen for differentiation inducers.

## Results

### A Haploid ESC Screen to Identify Genes that Promote Exit from Ground State Self-Renewal

To isolate and analyze mutant ESCs impeded in progression from self-renewal, we used a haploid reporter cell line (HRex1GFPd2) in which a destabilized version of GFP is expressed from the endogenous Rex1 (*Zfp42*) locus (Wray et al., 2011). Rex1 expression is tightly linked to naive pluripotency and is rapidly lost at the onset of differentiation. Importantly, lack of Rex1 is inconsequential for ESCs (Masui et al., 2008) and HRex1GFPd2 reporter ESCs lacking Rex1 protein contribute extensively to chimeras (Leeb et al., 2012). After withdrawal of 2i/LIF, GFP is substantially downregulated by 48 hr and <1% of cells remain positive by 72 hr, similar to the profile observed for homozygous Rex1GFPd2 ESCs derived from diploid embryos (Figure S1A available online) and previously described for heterozygous Rex1GFPd2 cells (Wray et al., 2011). Therefore, absence of Rex1 does not overtly alter expression of the reporter, indicating that the HRex1GFPd2 ESC line is a suitable tool to monitor exit from ground state self-renewal.

**Figure 1:**
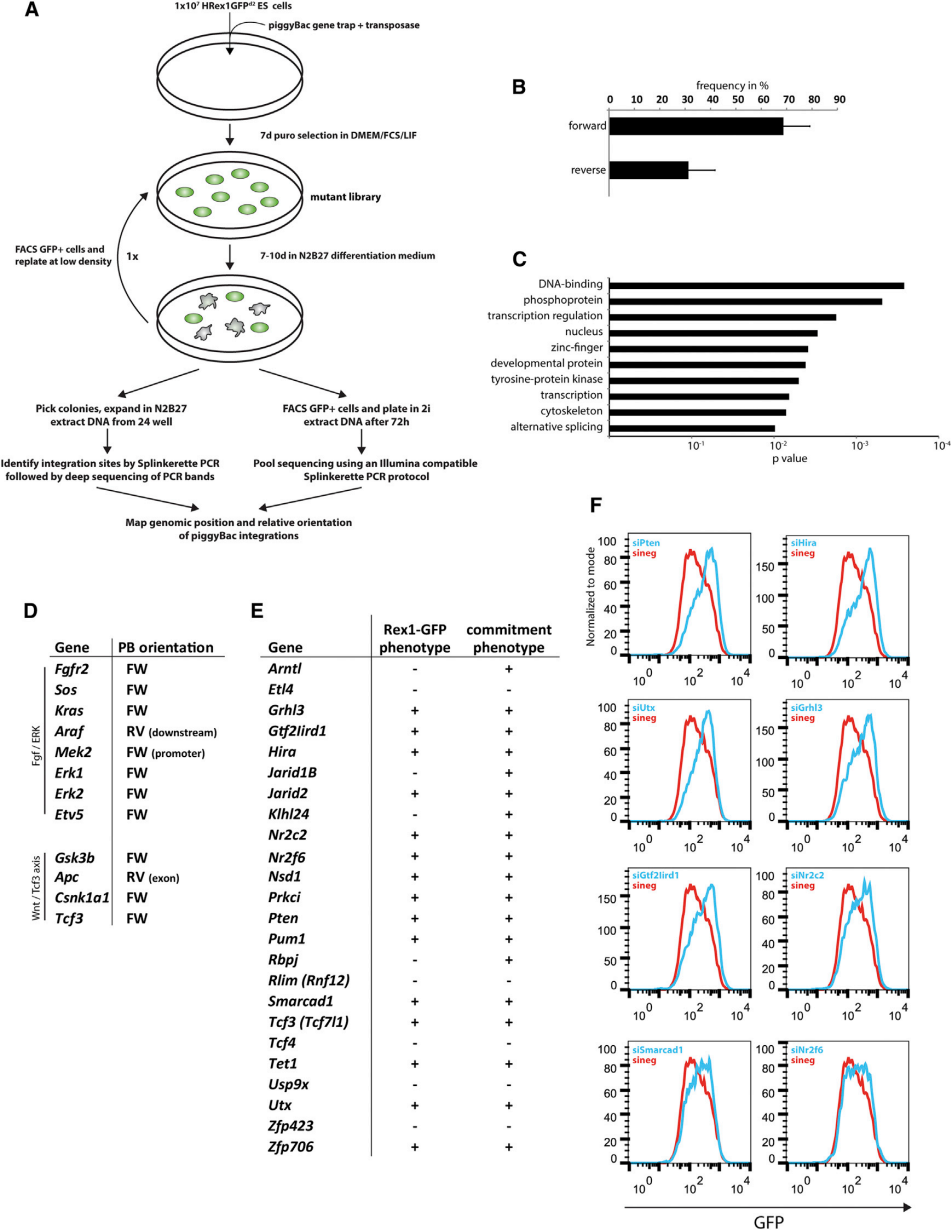
Screen for Resistance to Differentiation Using Haploid Rex1GFPd2 Reporter ESCs (A) Screening strategy used to identify regulators of the entry into differentiation. (B) Proportion of reads in forward (FW) and reverse (REV) orientations within transcription units. Mean and standard deviation between all screens is shown. (C) GO keyword analysis of all screen hits satisfying cutoff criteria. (D) Gene list showing members of the Fgf/ERK pathway and the β-catenin/Tcf3 axis identified in at least one screen. (E) List of 24 candidate genes that were subjected to siRNA validation. A plus sign denotes confirmation of a defect in the indicated assay. (F) siRNA knockdown of a subset of candidate genes in diploid Rex1GFPd2 ESCs 24 hr after withdrawal of inhibitors. See also Figure S1.

To produce a library of mutagenized ESCs, we used a piggyBac (PB) transposon as a vehicle for delivery of a gene trap cassette (Figures 1A and S1B). PB transposons have less bias toward certain genomic hot spots than retroviral vectors (Wang et al., 2009). At electroporation HRex1GFPd2 ESCs were confirmed to be more than 90% haploid by propidium iodide staining. Diploidization proceeds inexorably during culture (Leeb et al., 2012). Therefore mutagenesis of a haploid genome will ultimately result in homozygous diploid mutant ESCs. For each electroporation we used 1 × 10^7^ ESCs with 5 μg 5′-PTK-3′ transposon and 20 μg PB transposase. This gave an average of one to four PB integrations per haploid genome. Integrations of the PB transposon into an active transcription unit in the appropriate orientation render cells resistant to puromycin (Figure S1B). In total we generated 11 independent mutant libraries, each of which contained between 4 × 10^3^ to 1 × 10^4^ colonies.

For induction of differentiation 1.8 × 10^6^ ESCs from each mutant pool were plated onto a 175 cm^2^ dish in N2B27 medium without LIF or 2i. This condition is highly permissive for differentiation driven by autocrine factors (Ying et al., 2003). After 7–10 days cells that retained Rex1GFP expression were purified by flow sorting and replated in the same conditions. After a further 7–10 days GFP-positive ESC colonies were collected (Figure 1A).

We adopted two complementary strategies to identify mutations associated with persistent self-renewal. First, almost 300 ESC clones from seven independent screens were manually picked. These clones were expanded in N2B27 medium and then transferred to 2i/LIF for 48 hr to confirm ground state ESC status. Genomic DNA was extracted from some 200 ESC clones. Importantly, a control population transfected with transposase alone produced very few colonies and none of 12 that were picked could be expanded in N2B27. This suggests that the screening system is robust and not compromised by background mutations in haploid ESCs. PB integration sites were mapped by Splinkerette PCR (Li et al., 2010). We introduced barcodes for each screen and then pooled libraries for massively parallel sequencing. An arbitrary cutoff was set at a minimum of 20 reads in at least one screen. We considered an integration site as potentially causal if it lay within a transcription unit in forward orientation or if the integration was detected in an exon or in the promoter region. Additionally, we reasoned that causative genes should be expressed at the onset of differentiation and set a transcription threshold of RPKM ≥ 5 after 2i withdrawal for 16 hr. Thereby we could map PB integrations in 68 genes (Table S1 available online).

However, clonal isolation and analysis are rate limiting. Therefore, in a second approach we collected all GFP-positive cells after the second round of N2B27 exposure and replated them in 2i/LIF for 48 hr. We then extracted DNA from the entire population (Figure 1A). Four screens were independently barcoded. In total approximately 2.5 × 10^4^ colonies were screened and expected to encompass at least an equivalent number of PB integrations. We chose parallel screens rather than one large mutant pool because detection of a gene in independent iterations of the same screen informs on likely significance. PB integration sites were mapped using a modified Splinkerette PCR protocol that directly generates fragments compatible with parallel sequencing on an Illumina platform (Li et al., 2011). In this way we were able to map integrations satisfying cutoff criteria in 251 genes (Table S1).

### Mutations Are Recovered in Multiple Components of Pathways Known to Promote ESC Differentiation

In both clonal and high-throughput approaches, we observed a strong bias toward integrations present in the forward orientation (Figure 1B). Gene ontology keyword analysis showed a significant enrichment in terms relating to transcriptional regulation and development (Figure 1C). The Fgf/Erk pathway (Kunath et al., 2007) and the GSK3/Tcf3 (a.k.a. Tcf7l1) axis (Wray et al., 2011) play central roles in the exit from ground state pluripotency. Reassuringly, we identified mutations in multiple components of both pathways (Figure 1D).

The phenotypic strength of a mutation can be gauged by independent appearances. However, due to the potential for PB transposon local hopping (Wang et al., 2008), multiple integrations at a given genetic locus within a single screen were considered as one hit. Threshold criteria for repeat appearance were fulfilled by 113 genes (Table S2). The most frequently recovered gene was *Tcf3*, identified in every screen. This is in line with compelling evidence for a central function for Tcf3 in the progression from pluripotency (Guo et al., 2011; Martello et al., 2012; Pereira et al., 2006).

We identified a number of mutations in other genes previously implicated in ESC differentiation. Notably, we found four independent integrations in the atypical protein kinase C (PKC) member *Prkci*. A recent study reported that broad-spectrum pharmacological inhibition of PKC can block ESC differentiation, though the authors did not implicate Prkci specifically nor investigate extended culture in the absence of serum (Dutta et al., 2011). We confirmed that the PKC inhibitor Gö6983 blocks ESC differentiation (Figures S1C–S1E). However, the high concentration (10 μM) required to suppress differentiation also compromises serum-free growth and cultures collapse within three passages, potentially due to off-target effects. We also identified four independent integrations in the tumor suppressor gene *Pten*. Loss of Pten in ESCs reduces differentiation (Sun et al., 1999). An integration in the *Rbpj* gene is consistent with evidence that Notch promotes neural commitment of mouse ESCs (Lowell et al., 2006). Furthermore, a number of epigenetic modifiers suggested to function in the stabilization of commitment were identified. Notably, we found integrations into genes encoding the Polycomb group proteins Suz12 and Jarid2 and the histone demethylases Utx (a.k.a. Kdm6a) and Jarid1B (a.k.a. Kdm5b). These factors may stabilize lineage-appropriate gene expression patterns during the commitment process (Landeira et al., 2010; Pasini et al., 2007; Schmitz et al., 2011; Wang et al., 2012).

To test further the reliability of the haploid screen, we employed two complementary assays using siRNA perturbation. We selected 24 genes for analysis based on predicted molecular function and on relative abundance of sequencing reads. Diploid biparental Rex1GFPd2 ESCs were transfected and differentiation was induced by withdrawal of inhibitors 24 hr later. First Rex1GFP levels were measured after a further 24 hr and used as proxy to estimate the proportion of ESCs remaining undifferentiated (Martello et al., 2012). Second, persistence of self-renewal was tested functionally after 72 hr in N2B27 by restoration to 2i/LIF. Parental ESCs are committed to differentiate at this time point and either differentiate or die in presence of 2i. In contrast, cells that fail to enter differentiation and retain ESC characteristics will proliferate and form alkaline-phosphatase-positive colonies. To ensure elimination of differentiated cells, blasticidin selection for cells retaining Rex1 expression was initiated 48 hr after exposure to N2B27 medium and maintained during culture in 2i/LIF. This assay provides a quantitative readout of the efficiency of commitment (Betschinger et al., 2013). By these two approaches we were able to confirm anticipated functionality of Tcf3, Pten, Utx1, Jarid1B, Jarid2, and Rbpj in efficient exit from self-renewal (Figures 1E, 1F, S1G, and S1H). In addition, knockdown of the histone H3.3 chaperone Hira, the SWI/SNF-like ATP-dependent chromatin remodeler Smarcad1, the nuclear receptors Nr2c2 and Nr2f6, the SET domain protein Nsd1, the basic helix-loop-helix protein Arntl, and the transcription factors Gtf2Iird1 and Grhl3 all elicited defects in exit from self-renewal.

Interestingly, depletion of candidate genes in 2i before induction of differentiation showed a slight increase in Rex1GFP expression in some cases, suggesting a fortification of the self-renewal network (Figure S1I).

siRNA transfection may give insufficient reduction for efficient phenotype validation, notably for enzyme targets. One of the genes that showed only a weak siRNA phenotype is *Prkci*, whose role was corroborated using a small molecule inhibitor as discussed above. A second is the methylcytosine hydroxylase *Tet1*, which we detected in four independent screens. Neither were reliably validated using siRNA pools. However, phenotypes were apparent for a subset of single siRNAs (Figure S1J).

In total, knockdown of 19 out of 24 transcripts yielded phenotypes in Rex1GFP downregulation, cell commitment, or both (Figure 1E). Overall, the detection of a large number of known effectors of differentiation and a high rate of candidate confirmation by siRNA knockdown testify to the reliability of this screening platform.

### Zfp706 Function Is Required for Timely Exit from Self-Renewal

**Figure 2:**
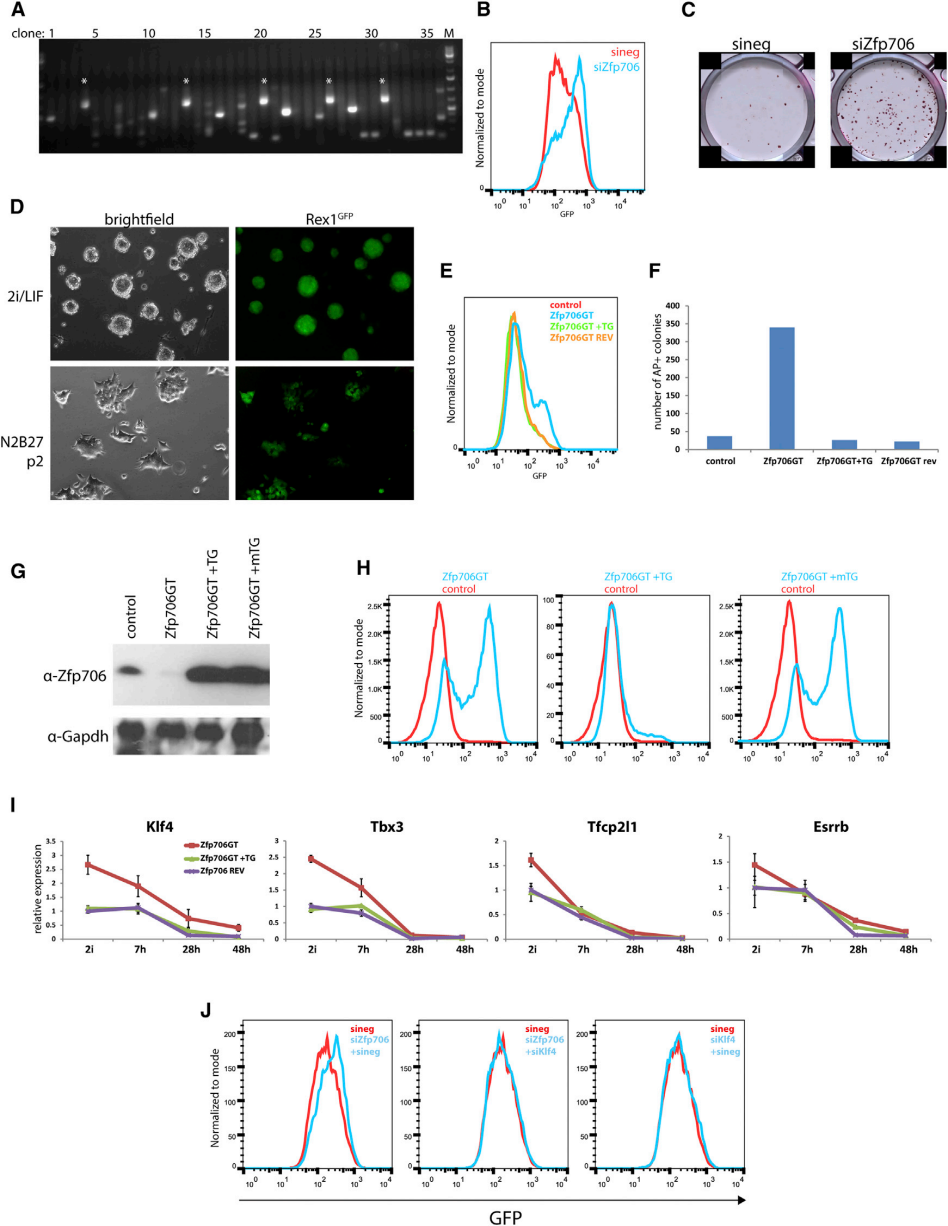
Zfp706 Is Required for Efficient Exit from Self-Renewal (A) Splinkerette PCR analysis of a clonal screen. Each lane corresponds to one clone; each band corresponds to one PB integration. Integrations in *Zfp706* are indicated with an asterisk. (B) Rex1GFPd2 profile 24 hr after induction of differentiation following knockdown of Zfp706 or control siRNA treatment. (C) Commitment assay after knockdown of Zfp706 confirms a role in the exit from self-renewal. Alkaline phosphatase (AP) staining was used to visualize ESC colonies. (D) Colony morphology and Rex1GFP expression in Zfp706GT ESCs in 2i/LIF and after two passages in N2B27. (E) Rex1GFP profiles of Rex1GFPd2, Zfp706GT, and clonal genetic revertant (Zfp706REV) or transgene-rescued Zfp706GT (Zfp706GT+TG) ESCs after 72 hr of differentiation. (F) Graph showing the number of AP-positive colonies generated by indicated ESC lines in a commitment assay. Colonies were counted using Image J software. (G) Western blot of indicated cell extracts probed with indicated antibodies. (H) Rex1GFP levels after 48 hr in N2B27 medium in control versus Zfp706GT, Zfp706GT +TG, and Zfp706 +mutant TG (mTG) ESCs. (I) Expression kinetics of indicated genes in a differentiation time course for Zfp706GT and rescue cell lines. Mean and SEM are plotted for each time point. Expression levels were normalized to Gapdh. (J) Rex1GFP profiles 24 hr after co-siRNA of Klf4 and Zfp706. See also Figure S2.

An integration in the gene encoding the small C2H2 zinc finger protein Zfp706 was recovered in multiple clones in one screen, indicating a strong phenotype (Figure 2A). The Zfp706 protein is only 76 amino acids in size. Functional importance is suggested by identical amino acid sequence between mouse, dog, rat, and human and high conservation in the vertebrate kingdom. Zfp706 protein is localized both in cytoplasmic and nuclear protein fractions (Figure S2A). We tested a role for Zfp706 in the exit from pluripotency by siRNA knockdown and observed interference with Rex1GFP downregulation (Figure 2B). Knockdown of Zfp706 also impeded functional exit from the ground state and cells retained the potential to self-renew in 2i medium after 72 hr in differentiation conditions (Figure 2C). A Zfp706 gene trap clone (Zfp706GT) could be recovered and exploited for further examination. Zfp706GT ESCs showed limited self-renewal in the absence of both LIF and 2i. They initially retained ESC colony morphology and the majority of cells maintained expression of GFP upon passaging (Figure 2D), whereas parental diploidized HRex1GFP ESCs cultured in parallel lost GFP after the first passage. Zfp706GT ESCs did eventually succumb to differentiation and few undifferentiated ESCs could be observed beyond passage four. Up to that point, however, ESC identity was retained as shown by contribution to term chimeras (Figure S2B).

We tested whether the *Zfp706* mutation was causal. First, Zfp706GT ESCs were transfected with Flpe recombinase to excise the gene trap cassette and restore function (Figure S1B). Second, we established Zfp706GT ESCs expressing a *Zfp706* transgene. Both strategies eliminated the delay in downregulation of Rex1 (Figure 2E) and rescued the functional deficiency in commitment (Figure 2F).

We generated a *Zfp706* transgene carrying two point mutations that abolish the function of the zinc finger by replacing both cysteines of the C2H2 domain with alanine. Expression of wild-type and mutant proteins was confirmed by western blotting (Figure 2G). Unlike wild-type Zfp706 protein, the point-mutated version failed to restore differentiation (Figure 2H). These data establish a role for Zfp706 in the exit from self-renewal that is dependent on its single annotated zinc finger domain. Interestingly, Zfp706 mRNA is present in self-renewing ESCs and increases only marginally at the onset of differentiation, which may suggest that its activity is dependent on partner proteins or posttranscriptional modification (Figure S2C).

**Figure 3:**
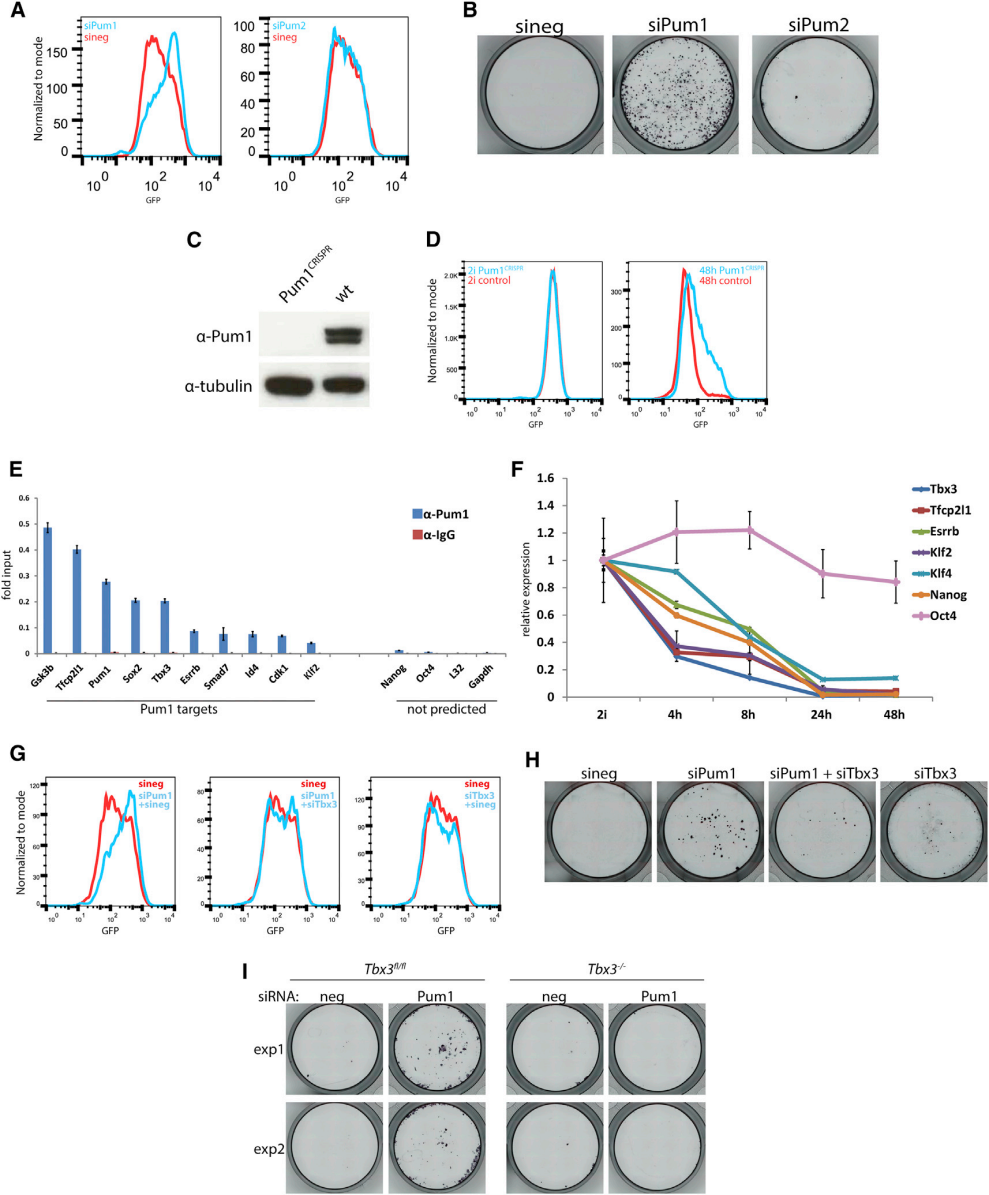
Pum1 Targets Naive Pluripotency Factor mRNAs during Exit from Self-Renewal (A) siRNA against Pum1, but not Pum2, results in delayed downregulation of Rex1GFP expression 24 hr after 2i withdrawal. (B) Pum1 knockdown results in a severe commitment defect. ESC colonies were stained with AP. (C) Western blot confirming absence of protein in CRISPR Pum1 mutant ESCs. (D) Impaired Rex1GFP downregulation in Pum1 mutant ESCs 48 hr after 2i withdrawal. (E) RIP experiment showing Pum1 binding to predicted Pum1 target mRNAs and control transcripts without Pum1 binding site. Rabbit IgG was used as negative control. Error bars show standard deviation between technical triplicates. Replicate experiments yielded equivalent results. (F) Gene expression profiles of indicated genes in a 48 hr N2B27 differentiation time course. Error bars represent standard deviation. Expression levels were normalized to Gapdh. (G) Rex1GFP profiles for cells transfected with indicated siRNAs 24 hr after 2i withdrawal. (H) Codepletion of Tbx3 restores differentiation in Pum1 siRNA transfected cells in a commitment assay. (I) *Tbx3*^fl/fl^ and *Tbx3*^*−/−*^ ESCs were transfected with the indicated siRNAs and tested in a commitment assay. AP staining was performed after 4 days in 2i/LIF. Duplicate experiments are shown. See also Figures S3 and S4.

To assess a potential function of Zfp706 in shutting down the pluripotency circuitry, we measured gene expression in ESCs deficient for Zfp706 and after restoration of Zfp706 function throughout a 48 hr differentiation time course (Figure 3I and Figure S2D). We detected a >2-fold upregulation of Klf4 and Tbx3, and a mild increase in Tfcp2l1 and Esrrb mRNA, in Zfp706GT ESCs in 2i. However, increased expression of Tbx3, Tfcp2l1, and Esrrb was not retained in differentiation conditions and vanished by 48 hr. In contrast, downregulation of Klf4 expression was impaired and 48 hr after the onset of differentiation, Klf4 expression persisted at around 40% of levels of controls in 2i.

Interestingly, the GFP-negative fraction of Zfp706GT ESCs 48 hr after withdrawal of 2i retained the ability to reestablish naive pluripotency (Figure S2E). The deficiency in locking in to differentiation was rescued in Zfp706REV cells. We found expression of Klf4 to be elevated in both the GFP-positive and -negative fractions of Zfp706GT cells. Strikingly, Klf4 levels were higher in the GFP-negative fraction of Zfp706GT cells than in the GFP-positive population of Zfp706REV cells.

Tcf3GT cells show a similar failure in commitment. However, they exhibit a different pattern of gene expression and maintenance of self-renewal capacity seems based mainly on persistent Klf2 and Esrrb expression after 2i withdrawal (Figure S2F) (Martello et al., 2013). Klf4 expression is only mildly affected in Tcf3GT cells, indicating that Zfp706 and Tcf3 promote differentiation by different mechanisms. Comparison of gene expression profiles between Zfp706GT and Zfp706REV ESCs in 2i using an Illumina MouseWG-6 v2 array showed little impact of loss of Zfp706 on global gene expression.

We tested genetic interaction between Zfp706 and Klf4 by concomitant knockdown. Deficiency in differentiation induced by Zfp706 knockdown was eliminated by Klf4 co-siRNA (Figure 2J).

Collectively these data indicate that Zfp706 function promotes stable repression of Klf4.

### Pum1 Promotes ESC Differentiation by Posttranscriptional Downregulation of the Naive Pluripotency Circuit

Gene trap insertions in the *Pum1* gene were identified in four independent screens (Table S2) and validated by siRNA (Figures 3A and 3B). Knockdown of the homolog Pum2 was without phenotypic consequence in either assay. For further validation we employed CRISPR-mediated mutagenesis in diploid Rex1GFPd2 ESCs and confirmed that *Pum*^*−/−*^ cells show delayed downregulation of the Rex1GFP reporter (Figures 3C and 3D).

Pum1 is a conserved RNA binding protein that inhibits translation and promotes degradation of target mRNAs (Chen et al., 2012) through binding 3′ UTRs at a highly conserved eight-nucleotide (8nt) motif (Galgano et al., 2008). Pum1 is constitutively expressed during early differentiation (Figure S3A).

Conservation of the Pum1 binding motif allowed us to predict targets among ESC transcripts with an RPKM value greater than five (Marks et al., 2012). We identified 1,674 transcripts that have at least one Pum1 binding site (Table S3). It may be expected that Pum1 targets associated with exit from self-renewal should be downregulated at the onset of differentiation. Among such genes, we noted three Pum1 binding sites clustered within a 75nt region in the 3′ UTR of the mRNA for the T-box transcription factor Tbx3. We also noted Pum1 binding sites in the 3′ UTRs of Tfcp2l1, Esrrb, and Klf2 mRNAs. These factors are each sufficient to maintain self-renewal capacity (Martello et al., 2013; Niwa et al., 2009). Additionally a Pum1 binding site was predicted in the 3′ UTR of Sox2.

We detected enrichment of Pum1 at predicted targets by RIP (Figure 3E). We examined gene expression after 2i withdrawal and found that Tbx3, Tfcp2l1, and Klf2 showed a marked reduction in transcript levels after only 4 hr (Figure 3F). Klf4 and Nanog did not show such an early response to 2i withdrawal. We then measured mRNA expression following depletion of Pum1 (Figures S3B and S3C). siRNA-mediated knockdown of Pum1 resulted in a mild increase in transcript levels of Tbx3, Tfcp2l1, and Sox2 in 2i and a marked delay in downregulation of Tbx3, Tfcp2l1, Klf2, Esrrb, and Sox2 during differentiation (Figure S3C). Klf4 mRNA, which does not contain a binding site for Pum1, did not react to depletion of Pum1. Interestingly, reduction in Nanog expression is also delayed following Pum1 siRNA transfection. This may reflect the connectivity within the pluripotency circuit whereby Pum1 targets directly regulate transcription of Nanog (Martello et al., 2013; Niwa et al., 2009).

The preceding data are consistent with a function of Pum1 in promoting posttranscriptional reduction of multiple members of the core pluripotency transcription factor network. To test for genetic interaction, we focused on Tbx3 because Tfcp2l1, Klf2, or Esrrb loss of function severely compromises ESC self-renewal (Martello et al., 2013) (Figure S4A). We observed restored Rex1GFP downregulation and functional commitment upon simultaneous knockdown of Pum1 and Tbx3 (Figures 3G and 3H). By comparison, depletion of Tbx3 did not restore differentiation in cells transfected with Tcf3 siRNA (Figure S4B). We also assessed the impact of Pum1 knockdown in Tbx3 null ESCs generated by serial gene targeting and conditional deletion (Figures S4C and S4D). Pum1 depletion reduced differentiation in *Tbx3*^fl/fl^ ESCs but had no effect on *Tbx3*^*−/−*^ cells (Figure 3I). Together, these data indicate that Pum1 facilitates the exit from the ground state by accelerating removal of major elements of the naive pluripotency circuitry.

## Discussion

Mutagenesis of haploid ESCs combined with deep sequencing provides unprecedented opportunities to dissect genetic pathways underlying biological events in mammalian cells. We explored the potential of transposon mutagenesis in haploid ESCs to annotate gene functions required for expedient entry of pluripotent cells into differentiation. This screen identified a gene set including known actors and many new candidates. Overall we mapped insertions in 311 genes. Some will be passengers because clones have up to four integrations. Therefore we employed a strategy of multiple parallel screens. Contribution of secondary sensitizing mutations is unlikely in cases of independent detection. Stringent siRNA assays in biparental cells provided rapid independent validation. The high confirmation frequency establishes that the screen detects genes that singularly affect the rate and efficiency of departure from the self-renewing ground state.

The present screen was carried out in the absence of exogenous inducers to provide a permissive context sensitized to mutations in a broad range of commitment effectors. Consistent with prior knowledge we recovered numerous elements in the Fgf/Erk signaling cascade and the GSK3/Tcf3 axis and provided genetic evidence to substantiate a role for atypical PKC family members (Dutta et al., 2011), pinpointing Prkci as the main effector. We also retrieved a number of transcription factors and several chromatin modifiers, including PRC2 components, histone demethylases, and Tet1. In addition we found the histone H3.3 chaperone Hira, which facilitates nucleosome exchange and can thereby alter regulatory sequence accessibility. Interestingly, Hira-mediated H3.3 deposition has been linked to the recruitment of PRC2 to its target sites (Banaszynski et al., 2013). Thus multiple mechanisms appear to be engaged to restructure chromatin and consolidate the rewiring of gene expression circuitries during the commitment process.

Factors not previously implicated in pluripotent cell biology were also unveiled. Among these the small zinc finger protein Zfp706 attracts attention because it is evolutionarily highly conserved. The role for Zfp706 appears to be focused on stable shutdown of mainly Klf4. The molecular mechanism of Zfp706 action remains to be elucidated. The single Zinc finger is unlikely to confer specific high affinity DNA binding and Zfp706 does not contain any other characterized domain to mediate DNA binding or other function. Identifying interaction partners is therefore likely to be key to elucidating how Zfp706 represses Klf4.

In contrast, the activity of the RNA binding protein Pum1 is well characterized although not previously linked to pluripotency. Pum1 inhibits translation and promotes degradation of target mRNAs. In ESCs Pum1 targets include prominent members of the naive pluripotency circuit. Differentiation is initiated by loss of a set of transcription factors that are specific to the naive state and underpin self-renewal (Nichols and Smith, 2012). Transcripts for the majority of these transcription factors are bound by Pum1. Strikingly, we found that, with the exception of Esrrb, Pum1 target mRNAs are downregulated more rapidly than other naive factors. This suggests that Pum1 may act as a circuit dampener that constrains and potentially can extinguish much of the self-renewal machinery. Pum1 binds to its target mRNAs in 2i. Thus two modes of action are possible. Pum1 may be wholly or largely inactive during self-renewal and triggered into action at the onset of differentiation. Reported protein interaction of Pum1 with Nanog is noteworthy in this regard (Gagliardi et al., 2013). Alternatively, Pum1 may act constitutively to reduce mRNA half-life, with a profound impact when transcription is reduced.

**Figure 4:**
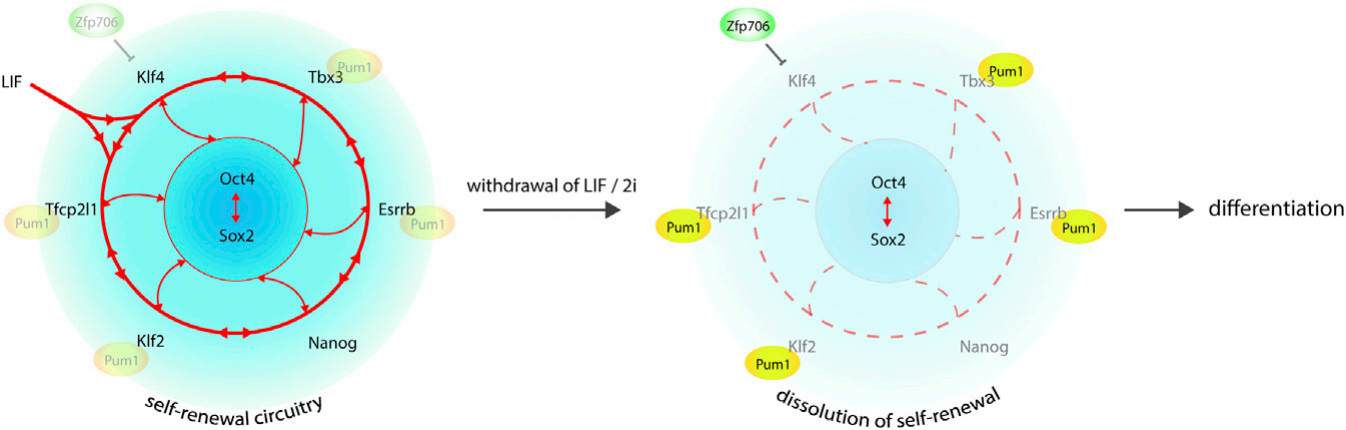
Zfp706 and Pum1 Action in Dismantling of the Naive Pluripotency Circuit (A) Pum1 binds to naive pluripotency factor mRNAs in self-renewing ESCs, targeting them for rapid elimination at the onset of differentiation. Zfp706 has a more selective effect by driving full repression of Klf4.

Developmental progression from ground state pluripotency entails dissolution of a transcription factor circuit that is highly recursive and robust. The mechanisms for dismantling this interconnected circuitry are poorly understood. Ffg4/Erk signaling leads to reduced expression of Nanog (Hamazaki et al., 2006) while Tcf3 promotes entry into differentiation by repression of Nanog, Klf2, and, in particular, Esrrb (Martello et al., 2012; Pereira et al., 2006). Cytoplasmic sequestration of the transcription factor Tfe3 by folliculin also causes downregulation of Esrrb (Betschinger et al., 2013). Pum1 targeting of multiple transcription factors and establishment of stable silencing of Klf4 by Zfp706 constitute further avenues of attack on the naive pluripotency circuit (Figure 4). Overcoming the extensive interconnectivity of the ESC transcription factor circuitry to enable differentiation thus appears to be achieved through parallel and overlapping mechanisms that in combination extinguish ESC identity. Future studies will determine the extent to which other candidates identified in our screen may represent additional failsafe mechanisms that act in concert with Tcf3, folliculin, Erk, Prkci and the newly discovered actions of Zfp706 and Pum1 to guarantee timely and efficient exit from the ground state. Annotating these mechanisms may also provide insight into stabilizing naive pluripotency in other species.

## Experimental Procedures

### Cell Culture and Generation of Transgenic Cell Lines

Haploid Rex1GFPd2-IRES-BSD (Rex1GFPd2) ESCs were cultured on 0.2% gelatine in 2i medium (NDiff B27 base medium, Stem Cell Sciences, cat. SCS-SF- NB-02, supplemented with 1 μM PD0325901, 3 μM CHIR99021, and 20 ng/ml LIF) as described (Ying et al., 2008).

### Differentiation Screen

To generate mutant pools, 10^7^ haploid ESCs were electroporated using a GenePulser Xcell (270 V, 500 μF, Biorad) with 5 μg 5′-PTK-3′ plasmid and codon optimized mPB transposase. Selection with puromycin was started 36 hr later. After 7 days, cells were plated at a density of 10^4^ cells/cm^2^ in N2B27 medium in the absence of LIF or inhibitors to allow differentiation. After 7–10 days in differentiation conditions, GFP-positive cells were sorted and replated at low density. After culture in N2B27 medium for a further 7–10 days, ESC clones that failed to differentiate were isolated and subject to further analysis.

### siRNA Transfections

siRNAs used are listed in Table S4. Transfection was performed using Dharmafect 1 (Dharmacon, T-2001-01) in a reverse transfection protocol.

### Splinkerette PCR and Illumina Library Preparation

gDNA was extracted and Splinkerette PCR was performed as described previously (Li et al., 2010). For amplification of transposon integration sites in a pool of cells, an adapted Splinkerette PCR protocol was employed (Li et al., 2011). Primer and adaptor sequences are listed in Table S4.

## Author Contributions

M.L. designed the study and performed and interpreted experiments. S.D. performed bioinformatical analysis. M.P. prepared transposon libraries. H.N. generated Tbx3-deficient ESCs. A.S. supervised the study and wrote the paper with M.L.

## Supplemental Information

Document S1. Supplemental Experimental Procedures and Figures S1–S4

Table S1. Screen Hits Fulfilling Cutoff Criteria in Clonal or High-Throughput Screen, Related to Figure 1

Table S2. Genes Fulfilling Criteria for Multiple Appearances, Related to Figure 1

Table S3. 3' UTRs of Expressed Genes Harboring a Pum1 Binding Site, Related to Figure 3

Table S4. Primers and siRNAs Used in the Study, related to Experimental Procedures
