# Supplementary material for: Genetic Exploration of the Exit from Self-Renewal Using Haploid Embryonic Stem Cells
Source: Cell Stem Cell. 2014 Mar 6;14(3):385–93. doi: 10.1016/j.stem.2013.12.008 (PMC3995090; doi:10.1016/j.stem.2013.12.008)
Supplement: Document S1. Supplemental Experimental Procedures and Figures S1–S4 [file mmc1.pdf]

Cell Stem Cell, volume *14*

Supplemental Information

Genetic Exploration of the Exit from Self-Renewal Using Haploid Embryonic Stem Cells

Martin Leeb, Sabine Dietmann, Maike Paramor, Hitoshi Niwa, and Austin Smith

Inventory of Supplemental Information

Supplemental Figures and Tables

Figure S1, related to Figure 1

Figure S2, related to Figure 2

Figure S3, related to Figure 3

Figure S4, related to Figure 3

Table S1, related to Figure 1

Table S2, related to Figure 1

Table S3, related to Figure 3

Table S4, related to Experimental procedures

Supplemental Experimental Procedures

Supplemental References

Figure S1, related to Figure 1 - Validation of the haploid screen

(A) Kinetics of GFP downregulation in haploid (HRex1GFPd2) vs biparental diploid homozygous Rex1GFPd2 (Rex1GFPd2) ES cells after withdrawal of 2i/LIF. (B) Schematic representation of the piggyBac (PB) transposon used in this study after productive integration into a transcription unit. Transcription is captured via the splice acceptor and terminated at the polyA site of the gene trap cassette. Flp recombinase mediated excision of the gene trap vector followed by negative selection (GCV) restores a wild type allele. (C-E) Flow profiles measuring GFP levels in Rex1GFP ES cells at different time points after 2i withdrawal treated with various concentrations of Gö6983. (F) Plot showing cell numbers relative to DMSO after treatment with different doses of Gö6983, 72 hours after plating ES cells in N2B27 at low density. Error bars represent standard deviation between two biological replicates. (G,H) Commitment assay determining the phenotype of candidate genes by siRNA mediated depletion. Alkaline Phosphatase staining was used to detect ES cell colonies. (I) FACS blots showing Rex1GFP levels after transfection with indicated siRNAs 24 hours after transfection in 2i. (J) Commitment assay using deconvoluted Tet1 and Prkci siRNAs.

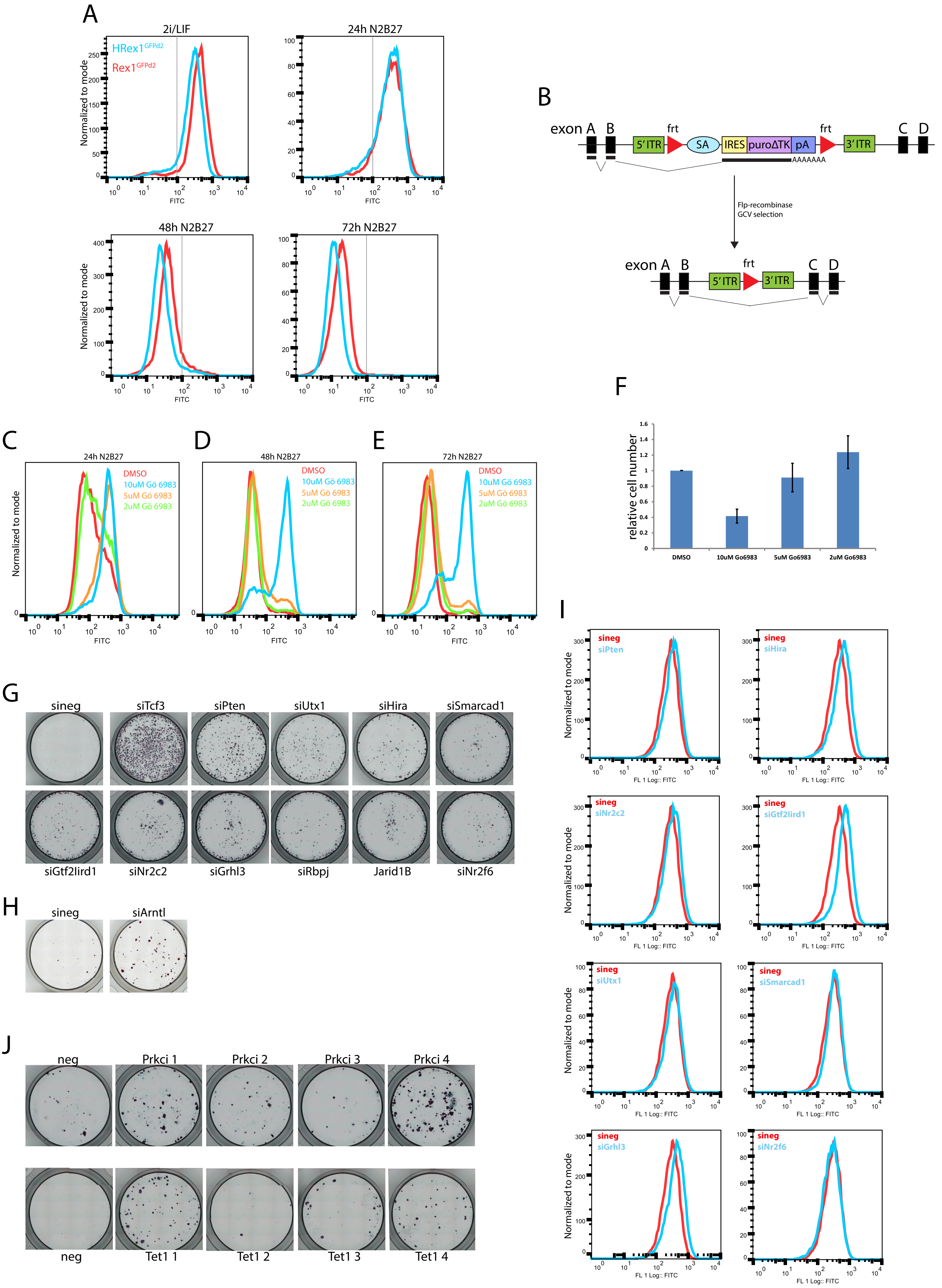

Leeb et al. Supplementary Figure 1 - relates to Figure 1

Figure S2, related to Figure 2 - Zfp706 is required for efficient exit from self-renewal

(A) Nuclear and cytoplasmatic fractions extracted from ES cells probed with indicated antibodies. Zfp706 protein is detected in both fractions. (B) Chimeras generated from Zfp706GT ES cells at passage four in N2B27 medium after blastocyst injection into C57BL/6 host embryos. Four out of 15 pups showed coat colour chimerism. In a control experiment with Zfp706GT ES cells grown in 2i, 3 out of 5 pups showed chimerism of a similar grade. (C) Expression kinetics of Zfp706 in a 48 hour time course. (D) Gene expression analysis of indicated genes over a 48 hour differentiation time course. (E) AP staining showing result from a replating GFP positive and negative populations after 48 hours of differentiation into 2i/LIF medium on gelatine. Zfp706GT and Tcf3GT ES cells retain the potential to self-renew in the GFP negative population. (F) qPCR analysis of indicated genes in fractions from (E).

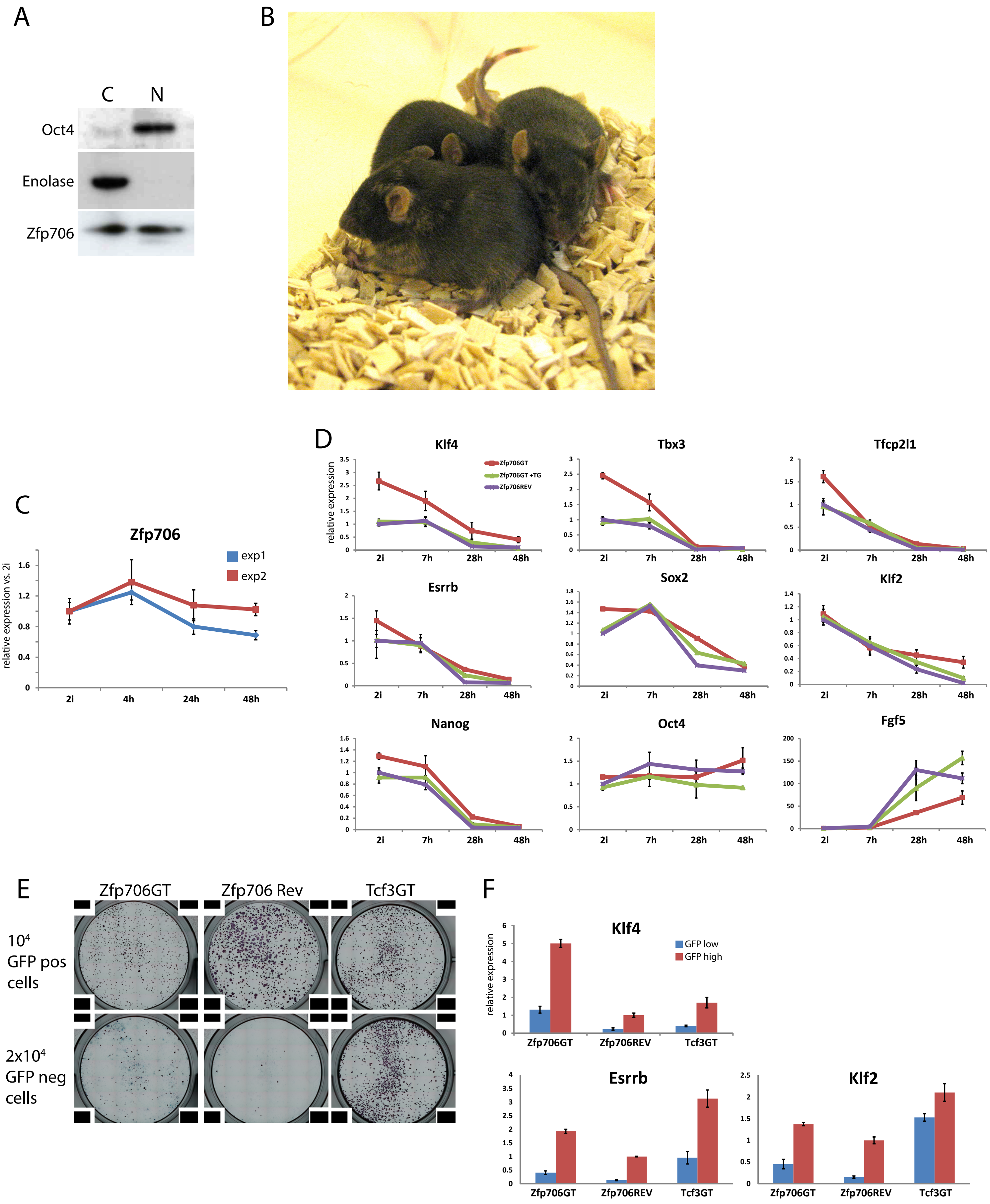

Leeb et al. Supplementary Figure 2 - relates to Figure 2

Figure S3, related to Figure 3 - Pum1 targets naïve pluripotency factor mRNAs during exit from self-renewal

(A) Expression kinetics of Pum1 in a 48 hour time course. (B) Expression of indicated genes in 2i after transfection with either negative siRNA (blue) or siRNA targeting Pum1 (red).

(C) qPCR analysis of indicated genes after transfection with either negative control orPum1 siRNA. Error bars represent standard deviation between replicates. Gapdh was used to normalise expression.

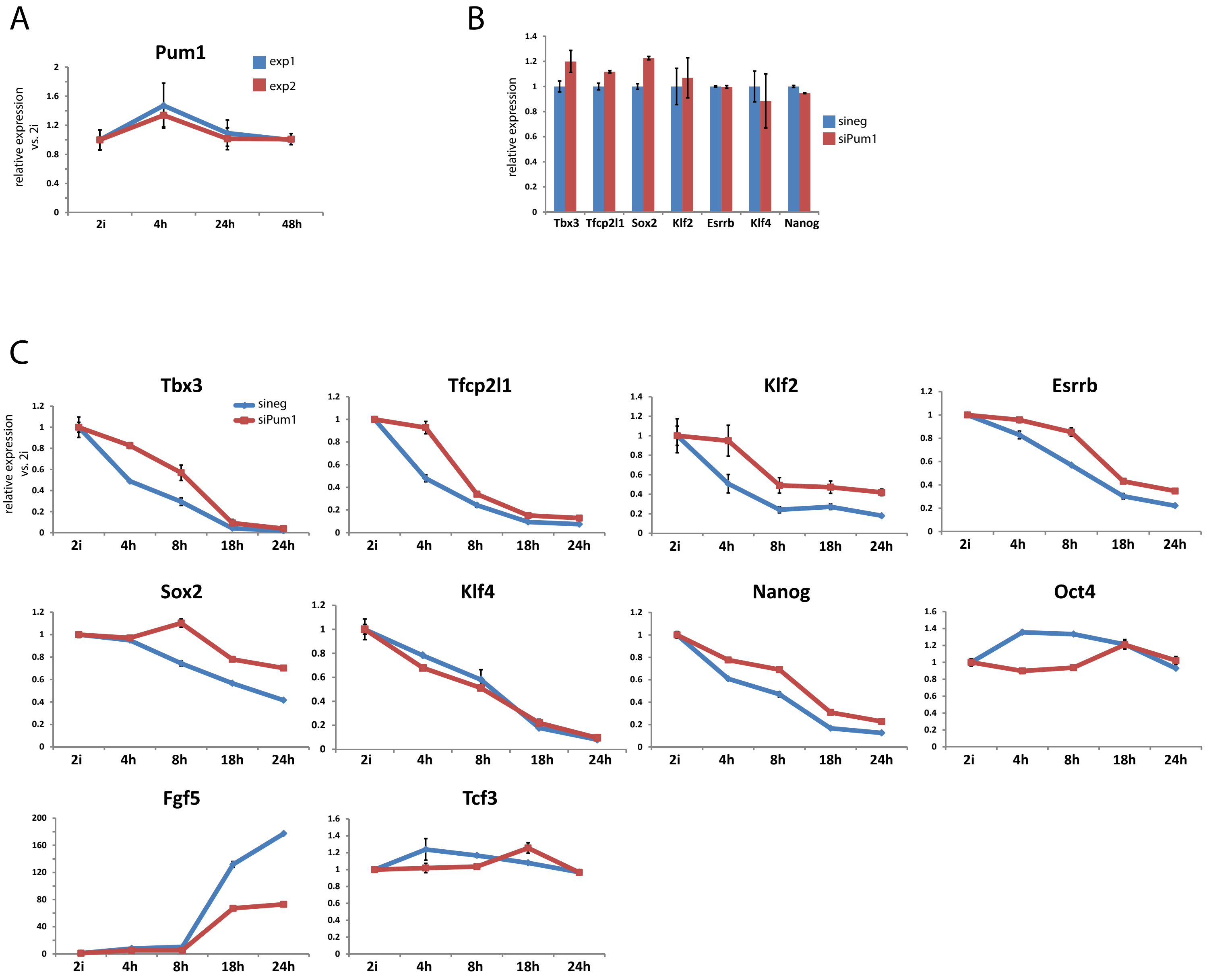

Leeb et al. Supplementary Figure 3 - relates to Figure 3

Figure S4, related to Figure 3 – Pum1 targets naïve pluripotency factor mRNAs during exit from self-renewal

(A) Knockdown of Tfcp2l1, Klf2, Nanog and Esrrb in 2i medium leads to a pronounced loss of Rex1GFP expressing cells compared to control 48h after siRNA transfections. (B) Concomitant knockdown of Tcf3 and Tbx3 does not rescue the differentiation deficiency observed upon Tcf3 single knockdown. (C) Schematic overview of the conditional gene targeting used to disrupt the Tbx3 locus. (D) qPCR analysis using primers spanning the deleted exons two to four of Tbx3 confirms excision of loxP flanked sequence. Oct4 expression is unaffected. Expression levels were normalised to Gapdh. Error bars represent standard deviation between replicates.

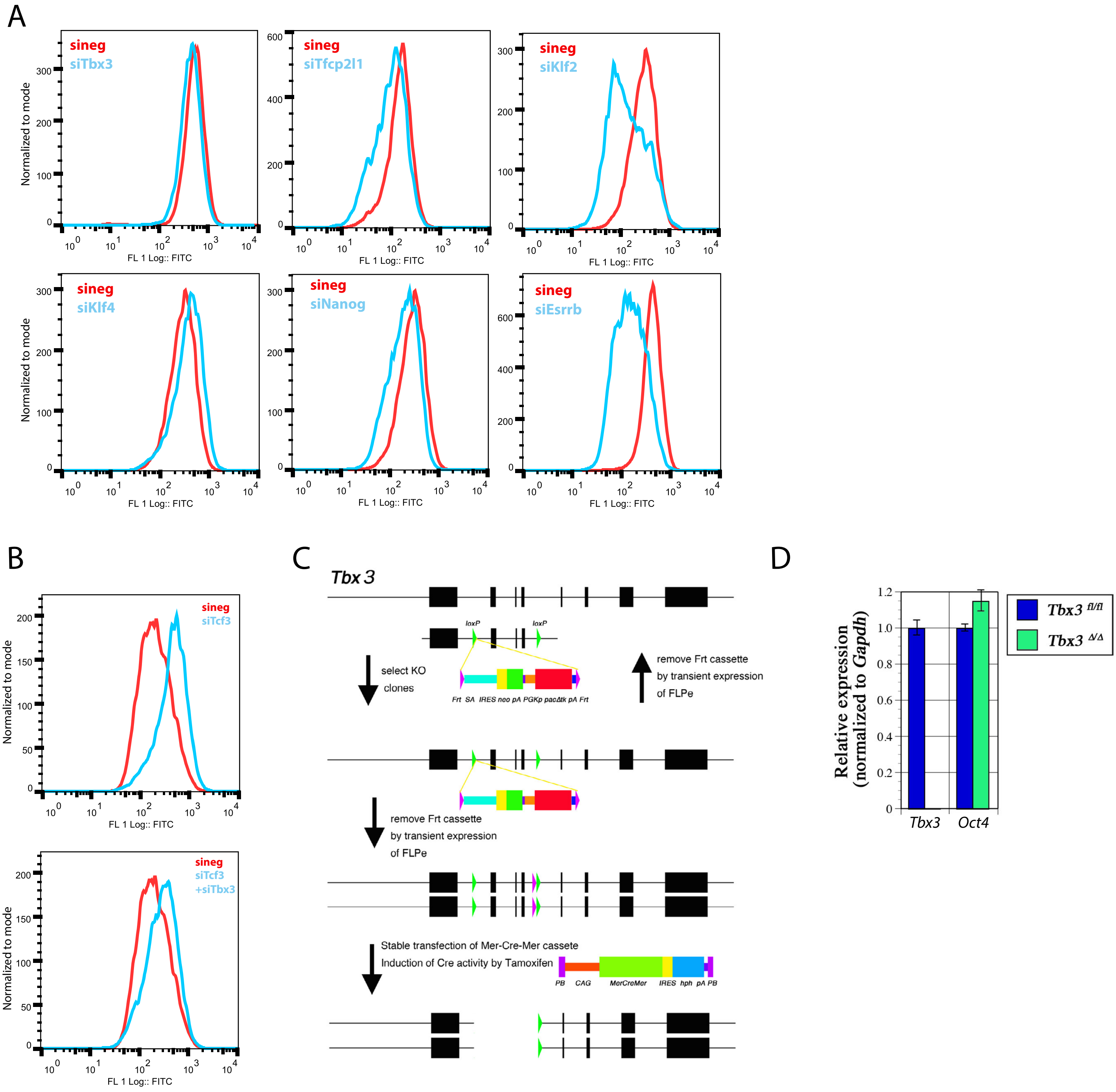

Leeb et al. Supplementary Figure 4 - relates to Figure 3

Table S1, related to Figure 1

Tabs 1 to 3 show genes identified in 7 clonal and 4 high throughput screens. All genes identified in forward or reverse orientation with a total read count of above 5 are shown. RPKM gene expression values throughout a 24 hour differentiation time course and the relative position of the integration in the gene body are indicated. The summary tab shows in separate columns all genes identified in the clonal and high throughput screens.

Table S2, related to Figure 1

Genes fulfilling cut-off criteria (>= 20 reads AND in forward orientation OR in exon OR up to 500bp upstream of TSS) in at least one screen, and present in at least one more screen with cutoff (>=5 reads AND in forward orientation OR in exon OR up to 500bp upstream of TSS). All genes need to fulfil the expression cut-off of RPKM >=5 16 hours after the onset of differentiation.

Table S3, related to Figure 3

List shows RefSeq genes with potential Pum1 target sites and an RPKM expression value in ES cells of above 5 (Marks et al., 2012). The numbers of predicted Pum1 target sites in 3'UTRs are indicated. RPKM values in 2i are shown.

Table S4, related to Experimental Procedures

List of primers and siRNAs used in this study

Supplemental Experimental Procedures

Cell Culture and generation of transgenic cell lines

Haploid Rex1GFPd2-IRES-BSD (Rex1GFPd2) ES cells were cultured on 0.2% gelatine in 2i medium (NDiff B27 base medium, Stem Cell Sciences Ltd, cat. SCS-SF- NB-02, supplemented with 1 µM PD0325901, 3 µM CHIR99021 and 20ng/ml LIF) as described (Ying et al., 2008). Gö6983 was obtained from Sigma (G1918). Derivation of the HRex1GFPd2 haploid ES cell line was described previously (Leeb et al., 2012). Selection for gene trap mutants was performed in FCS containing ES cell medium (DMEM high glucose, 15% FCS , 2 mM L-Glutamine, Pen/Strep, NEAA, 1mM Sodium Pyruvate (all from Life Technologies), 100mM β-Mercaptoethanol (Sigma) and 10ng/ml LIF.

Cell sorting for DNA content was performed after staining with 15 µg/ml Hoechst 33342 (Invitrogen) on a MoFlo flow sorter (Beckman Coulter) as described (Leeb and Wutz, 2011). The haploid 1n population was purified and plated in 2i/LIF medium supplemented with 2% FCS to increase attachment. Analytic flow profiles of DNA content were recorded after fixation of the cells in ethanol, RNase digestion and staining with propidium iodide (PI) on a CyanADP analyser (Beckman Coulter).

For Zfp706 overexpression the full length coding sequence of Zfp706 was cloned into a PB based vector to generate the pCAG-Zfp706-pA:PGK-hygro-pA plasmid. The plasmid was linearized with SalI and Lipofectamine 2000 (Life Technologies) was used as described (Betschinger et al., 2013) to transfect Zfp706GT ES cells in order to generate a pool of Zfp706 overexpressing cells (Zfp706GT +TG) after selection with 150ug/ml Hygromycin B. Genetic reversion of the Zfp706 GT mutation was accomplished by transient transfection of a circular CMV-FlpE vector (Figure S1B). To select for the excision of the gene trap cassette, cells were exposed to 2 µM Ganciclovir (Sigma) for 7 days. Twelve surviving colonies were picked and expanded in 2i/LIF medium. Excision of the genetrap cassette was confirmed by PCR (data not shown). Three clones were chosen for further analysis to confirm reversion of the differentiation phenotype.

For site directed mutagenesis, complementary DNA oligo primers carrying the 2C to 2A substitution were used to amplify the pCAG-Zfp706-pA:PGK-hygro-pA plasmid in a PCR reaction. The template wild type copy was digested with DpnI (NEB). Thereafter, the linear PCR product was electroporated into JM109 electrocompetent bacteria. The presence of the mutated sequence was confirmed by Sanger sequencing. Images were obtained on a Zeiss Axioplan microscope. For CRISPR mutagenesis a construct containing the guide RNA sequence 5'-GAGCAAACATCGATGGCCTAC-3' targeting the first exon of Pum1 was co-transfected with two further plasmids encoding Cas9 nuclease and dsRed using Lipofectamine 2000. gRNA design was performed using online resources provided at www.genome-engineering.org (Cong et al., 2013). Single dsRed positive cells were deposited by FACS 48 hours after transfection into a 96 well plate.

All animal experiments were performed in accordance with University guidelines and covered by UK Home Office licenses.

Differentiation screen

To generate mutant pools, 10^7 haploid ES cells were electroporated using a GenePulser Xcell (270 V, 500 µF, Biorad) with 5 µg 5'-PTK-3' plasmid and codon optimised mPB transposase (both obtained from the Wellcome Trust Sanger institute) (Cadinanos and Bradley, 2007). ES cells in the presence of FCS and LIF show more promiscuous gene expression than ES cells in 2i (Marks et al., 2012). Therefore, to maximize the complexity of the gene trap pools we performed puromycin selection in FCS and LIF. Selection with puromycin (1 µg/ml) was started 36 hours later. After 7 days, cells were counted on a ViCell XR (Beckman Coulter) and plated at a density of 10^4 cells/cm^2 in N2B27 medium in the absence of LIF or inhibitors to allow differentiation. After 7 to 10 days in differentiation conditions, GFP positive cells were isolated on a MoFlo sorter (Beckman Coulter) and plated at a density of 10^4 cells/cm^2 onto an appropriately sized gelatinized tissue culture dish. Cells were cultured in N2B27 medium for a further 7 to 10 days. In the clonal screening approach a total of approximately 300 ES cell like colonies were manually picked from 7 independent screens based on

morphological criteria and expanded in N2B27 medium until they could be plated into a 12 well plate. Gene trap clones were grown for 48 hours in 2i/LIF medium to select for ground state pluripotent ES cells before extracting gDNA using the Genetra Puregene Cell Kit (Quiagen). In the high throughput approach, GFP positive cells were flow cytometrically purified after the second round of N2B27 differentiation. GFP positive ES cells were expanded in 2i/LIF medium for 48h before extraction of genomic DNA.

Splinkerette PCR and Illumina library preparation

gDNA was extracted using the Gentra puregene cell kit (Quiagen) and Splinkerette PCR was performed as described previously (Li et al., 2010). Genomic fragments flanking the PB integration site were amplified using specific primers in the PB 5' and 3' terminal repeats for four clonal screens (Li et al., 2010). Splinkerette PCR bands for three further clonal screens were generated using only PB 3' specific primers. To generate a library for deep sequencing PCR fragments were end repaired (NEB Next end repair module) followed by A-tailing (NEB Next A-tailing module). Thereafter, Illumina compatible adapters (NEXTflex, Bio Scientific) were ligated to uniquely barcode each clonal screen. This was followed by 8 cycles of PCR to generate a sequencing library. To reduce PCR bias we pooled fragments generated by KAPA HiFi and QIAGEN Multiplex polymerases in equal amounts.

For amplification of transposon integration sites in a pool of cells an adapted Splinkerette PCR protocol was employed (Li et al., 2011). Primer and adapter sequences are listed in Table S4. In brief, genomic DNA was sheared, end repaired and A tailed followed by ligation of Splinkerette adapters. Transposon integration sites were amplified in a nested PCR approach using KOD hot start polymerase (Merck, 71086). In the secondary PRC, Illumina specific sequences were introduced. Each of the four high throughput libraries was uniquely tagged with an 8nt barcode. Sequencing was performed on Illumina MiSeq or HiSeq2000 machines at the EMBL Core Sequencing facility and the Eastern Sequence and Informatics Hub.

Bioinformatic analysis

Read pairs were trimmed of adapters and PB tags before mapping the genomic fragments to the genome as follows: For the clonal libraries, the adapter sequence was removed from the 5' end of one mate with cutadapt (http://code.google.com/p/cutadapt). For the corresponding non-adapter mate (PB mate), the PB sequence was removed at the 5'end requiring an overlap of at least 15 nt. The adapter reads were further trimmed by PB sequence at the 3' end of the reads (at least 3 nt overlap). Genomic sequences were required to start with the Sau3AI recognition site GATC from the adapter end.

For PB mate reads that were too short for an alignment to the genome, the genomic sequences +/- 2 kb around the gene regions where the adapter reads matched was extracted, and the PB reads were aligned with bowtie at the extracted genomic regions requiring exact matches and selecting the closest match. “X” in the integration site column in clonal libraries 1 to 4 indicates that the integration site could not be exactly defined, but will be within the fragment size of the used library (100 to 300 bp).

For the high throughput libraries, the PB tag TAGGGTTAA was removed from the 5' end of the first mate with a custom script, and the adapter sequence was removed from the 3' end of the first mate requiring an overlap of at least 3 nt. For the corresponding mate, the PB sequence was removed from the 3'end.

Trimmed paired end reads were aligned to the mouse reference genome (mm10) with the bowtie2 software (http://bowtie-bio.sourceforge.net/bowtie2) allowing two mismatches and selecting unique alignments. In case of identification of an identical integration site in independent screens, we considered it as potential cross-contamination and assigned it to the library where it showed the largest number of reads. Only genes expressed 16 hours after the onset of differentiation in N2B27 with an RPKM greater or equal to 5 were considered (T.Kalkan, A.Smith, unpublished).

To predict potential Pum1 target mRNAs, 3'-UTR sequences of RefSeq genes were retrieved from the UCSC genome browser (mm10). The 3'-UTR sequences were searched for the presence of Pum1 motifs (TGTA[ATC]ATA) (Chen et al., 2012; Galgano et al., 2008) by PERL regular expression search.

siRNA transfections

siRNAs were obtained from Quiagen. siRNAs used are listed in Table S4. Transfection was performed using Dharmafect 1 (Dharmacon, cat. T-2001-01). Pools of at least two siRNAs were used if not otherwise indicated. Knockdown efficiency was confirmed to be higher than 60% in all cases. Cells were plated at a density of $1.5x10^4/cm^2$ onto gelatine coated 24 well plates in 2i medium without antibiotics. 24h after transfection the medium was changed to N2B27 after carefully washing with PBS. GFP profiles were recorded 24 to 48h later on a Cyan analyser. Live dead discrimination was performed using Topro-3.

To assay cell commitment cells were kept in N2B27 medium for 72h before adding 2i/LIF. Selection for Rex1 expressing cells using 5µg/ml Blasticidin (BSD) was initiated after 48h of differentiation. AP staining was performed after four days in 2i/LIF +BSD as described (Betschinger et al., 2013). Plates were imaged using an Olympus IX51, DP72 camera with CellSens software. Analysis was performed using ImageJ.

RNA and protein analysis

The RNeasy Kit (Quiagen) was used for extraction of total RNA. cDNA was transcribed from 0.5 to 1ug RNA using the Superscript III kit and oligo-dT primers. Real-time PCR was performed on a StepOnePlus machine (Applied Biosystems) using the Fast Sybr green master mix (Applied Biosystems). Expression levels were normalized to Gapdh. All experiments were performed at least

in biological duplicates if not otherwise indicated. Results are shown as mean and standard deviation if not otherwise indicated.

Gene expression in Zfp706GT and Zfp706 REV ES cells was assessed in biological triplicates on an Illumina MouseWG-6 v2.0 Expression BeadChip array. Sample processing and basic analysis was performed at Cambridge Genomic Services using standard protocols and Bioconductor's lumi package in R. Datasets are deposited in the GEO repository under the accession number GSE53194. Proteins were extracted with RIPA buffer (50 mM Tris, pH 7.4, 150 mM NaCl, 1 mM EDTA, 1% Tx-100, 0.1% SDS) supplemented with cOmplete Mini protease inhibitor cocktail (Roche). 3% milk was used for blocking. Antibodies used are Zfp706 (Santa Cruz, sc-87770), Enolase (Cell Signaling, 3810, 1:1000), Oct4 (Santa Cruz, sc-5279, 1:500) and GAPDH (Sigma, G8795, 1:2000).
For nuclear / cytoplasmic fractionation we used the NE-PER Nuclear and Cytoplasmic Extraction Reagent and followed the manufacturer's recommendation (Thermo, 78833).

RNA-IP

RNA-IP was performed using the Magna RIP RNA-Binding Protein Immunoprecipitation Kit (Millipore) according to the manufacturer's recommendations. Briefly, $2x10^7$ cells per IP were lysed by a freeze thaw cycle in hypotonic buffer. Immunoprecipitation was performed using Pum1 RIPAb antibody and anti-IgG (Millipore, 03-242) as control. Reverse transcription and qPCR was performed as above. Relative binding compared to input was calculated. Error bars show the standard error between technical duplicates. Two replicate experiments yielded equivalent results.

Generation of Tbx3 Knockout ES cells

Genomic DNA fragments of the *Tbx3* gene were amplified with KOD Fx DNA polymerase using the primer pairs attgcggccGCCTTTCAGACGTAGGCTGAGCTGAGGAG & attactagtCATTGGGGTCCTTAATAGACTTATTTC; attaCTAGTGTTTAAAACCACCGATTTAAGA &

aataCTAGTGAGTCACCAATGAACACTCTTC; aatactagtGTGGAGCTTGGCAATTGTGGAC & atcgatCACACCGATATTATCTGTAGAGATTCC . A gene targeting vector carrying Frt-SA-IRES-neo-pA-PGK-pacΔtk-pA-Frt was linearized and introduced into EB5 ES cells by electroporation followed by selection with 240 µg/ml of G418 and 1.0 µg/ml of puromycin for 8 days. Clones were picked and genotyped by PCR with KOD-Fx for both 5' and 3' ends. Targeted clones were expanded and transfected with pCAG-FLPe-IP by lipofection followed by selection with 1 µM gancyclovir for 8 days. Removal of the Frt cassete was confirmed by PCR. The second allele was then targeted with the same vector. Double targeted clones were expanded and stably transfected with a PB vector containing MerCreMer. To obtain deleted clones, cells were cultured with 4-hydroxytamoxifen for 2 days followed by clonal isolation. Loss of the floxed region was tested by PCR and absence of Tbx transcript confirmed by qRT-PCR.

Supplemental references

Betschinger, J., Nichols, J., Dietmann, S., Corrin, P.D., Paddison, P.J., and Smith, A. (2013). Exit from Pluripotency Is Gated by Intracellular Redistribution of the bHLH Transcription Factor Tfe3. Cell *153*, 335-347.

Cadinanos, J., and Bradley, A. (2007). Generation of an inducible and optimized piggyBac transposon system. Nucleic acids research *35*, e87.

Chen, D., Zheng, W., Lin, A., Uyhazi, K., Zhao, H., and Lin, H. (2012). Pumilio 1 suppresses multiple activators of p53 to safeguard spermatogenesis. Curr Biol *22*, 420-425.

Cong, L., Ran, F.A., Cox, D., Lin, S., Barretto, R., Habib, N., Hsu, P.D., Wu, X., Jiang, W., Marraffini, L.A., *et al.* (2013). Multiplex genome engineering using CRISPR/Cas systems. Science *339*, 819-823.

Galgano, A., Forrer, M., Jaskiewicz, L., Kanitz, A., Zavolan, M., and Gerber, A.P. (2008). Comparative analysis of mRNA targets for human PUF-family proteins suggests extensive interaction with the miRNA regulatory system. PloS one *3*, e3164.

Leeb, M., Walker, R., Mansfield, B., Nichols, J., Smith, A., and Wutz, A. (2012). Germline potential of parthenogenetic haploid mouse embryonic stem cells. Development *139*, 3301-3305.

Leeb, M., and Wutz, A. (2011). Derivation of haploid embryonic stem cells from mouse embryos. Nature *479*, 131-134.

Li, M.A., Pettitt, S.J., Yusa, K., and Bradley, A. (2010). Genome-wide forward genetic screens in mouse ES cells. Methods Enzymol *477*, 217-242.

Li, M.A., Turner, D.J., Ning, Z., Yusa, K., Liang, Q., Eckert, S., Rad, L., Fitzgerald, T.W., Craig, N.L., and Bradley, A. (2011). Mobilization of giant piggyBac transposons in the mouse genome. Nucleic acids research *39*, e148.

Marks, H., Kalkan, T., Menafra, R., Denissov, S., Jones, K., Hofemeister, H., Nichols, J., Kranz, A., Stewart, A.F., Smith, A., *et al.* (2012). The transcriptional and epigenomic foundations of ground state pluripotency. Cell *149*, 590-604.

Ying, Q.L., Wray, J., Nichols, J., Batlle-Morera, L., Doble, B., Woodgett, J., Cohen, P., and Smith, A. (2008). The ground state of embryonic stem cell self-renewal. Nature *453*, 519-523.
